# What works? Lessons from a pretrial qualitative study to inform a multi‐component intervention for refugees and asylum seekers: Learning Through Play and EMDR Group Traumatic Episode Protocol

**DOI:** 10.1002/jcop.22908

**Published:** 2022-06-14

**Authors:** Safa Kemal Kaptan, Betul Yilmaz, Filippo Varese, Panoraia Andriopoulou, Nusrat Husain

**Affiliations:** ^1^ School of Health Sciences, Manchester Academic Health Science Centre, Division of Psychology and Mental Health, Faculty of Biology, Medicine and Health The University of Manchester Manchester UK; ^2^ Complex Trauma and Resilience Research Unit Greater Manchester Mental Health NHS Foundation Trust Manchester UK; ^3^ Department of Psychology Manchester Metropolitan University Manchester UK

**Keywords:** asylum seekers, EMDR G‐TEP, intervention, mental health, parenting, pretrial, refugees

## Abstract

Almost half of the trials failed to recruit their targeted sample size of which 89% could be preventable. Successful implementation of mental health trials in a context of forcibly displaced individuals can be even more challenging. Mental health difficulties have the potential to impact parenting skills, which are linked to poor development in children, while parenting interventions can improve parents' mental health and parenting behaviors. However, the evidence on parenting interventions for refugees is limited. A parenting intervention, Learning Through Play Plus Eye Movement Desensitization and Reprocessing Group Treatment Protocol, has been designed to address parental mental health. This pretrial qualitative study, conducted with refugees, asylum seekers and professionals, aimed to explore their perceptions of the intervention and to identify barriers and recommendations for better engagement, recruitment, and delivery. Three themes were generated from thematic analysis: the content of the intervention, suggestions for improvement and implementation, and understanding the role of the facilitator. These themes provided insights into the issues that might predict the barriers for delivery of the intervention and offered several changes, including destigmatization strategies to improve engagement.

## 1 INTRODUCTION

According to a recent study, almost half of the trials conducted in the United Kingdom (44%) failed to recruit their targeted sample size due to several reasons related to trial design, content and delivery (Walters et al., 2017), of which 89% could be preventable (Briel et al., 2016). Successful implementation of mental health trials in a context of forcibly displaced individuals can be even more challenging due to difficulties in recruitment (Gabriel et al., 2017; Sulaiman‐Hill & Thompson, 2011), problems in gaining trust (Yelland et al., 2014), communication challenges (Burchill & Pevalin, 2012; N. K. Jensen et al., 2013), a different understanding of mental health (Feldmann, Bensing, & de Ruijter, 2007; Riggs et al., 2012), time constraints (Bennett & Scammell, 2014), high prevalence of dropout (Semmlinger & Ehring, 2021) and frequent resettlement and domestic duties (Robertshaw et al., 2017). Several studies have recommended conducting pretrial in‐depth qualitative studies with the target groups to identify potential challenges and to improve the chances of successful engagement by exploring the target groups' views on the proposed interventions at the design phase (Damschroder et al., 2009; Duggleby et al., 2020; Lewin et al., 2009; O'Cathain et al., 2013, 2014; Turner et al., 2019). Despite many researchers have also highlighted the importance of focusing on the needs of family members in the displacement context to inform and develop evidence‐based interventions for refugees and asylum seekers (RAS), this form of pretrial studies is rarely carried out (Eruyar et al., 2020; O'Cathain et al., 2013; S. M. Weine, 2011).

In the last decade, the scale of the refugee crisis has increased to the point that the greatest number of people in history have been forcibly displaced worldwide with fewer being able to return to their home countries. According to the UN High Commissioner for Refugees, there are currently around 26 million refugees globally, with half of them being children under the age of 18 (United Nations High Commission for Refugees & UNHCR, 2020). With this dramatic rise in the numbers of displaced individuals worldwide, the mental health of these groups has become a global concern. It is now well documented that RAS are at great risk of developing mental health difficulties due to forced displacement, traumatic events during war and flight, and adaptation difficulties during resettlement (Alpak et al., 2015; Steel et al., 2006). Systematic reviews have reported an increased prevalence of posttraumatic stress disorder, depression, anxiety, sleep disturbances, and severe disorders, including psychosis in RAS compared to the native population (Blackmore et al., 2020; Bogic et al., 2015; Fazel et al., 2005, 2012; Morina et al., 2018).

RAS may also face difficulties in supporting their children's emotional and physical development (Frounfelker et al., 2019; Sangalang et al., 2017). Parents may develop compromised caregiving behaviors as mental health difficulties, previous traumatic experiences, or daily stressors in the host country may become a catalyst for preexisting issues (Akesson & Sousa, 2020; Miller et al., 2020). Accumulating research has indicated that refugee and asylum seeker parents with posttraumatic stress disorder are more likely to show harsh parenting (Bryant et al., 2018), excessive control behaviors (Sim et al., 2018), maternal withdrawal (East et al., 2018), detachment symptoms and rejection of parental interactions (Eruyar et al., 2020), role‐reversal behaviors (Field et al., 2013), lack of involvement in children's development and insensitive, unstructured, hostile, and unresponsive attitudes toward their children (van Ee et al., 2012).

The linkage between maltreatment and the tendency for children to develop mental health difficulties is prevalent in RAS (Scharpf et al., 2020). Traumatic stress in parents is linked to increased depression, anxiety, insecure attachment, and behavioral problems in refugee children (Back Nielsen et al., 2019; Bryant et al., 2018; T. K. Jensen et al., 2015; Scharpf et al., 2019, 2020; Tay et al., 2020). On the other hand, parental engagement and family cohesion are factors that protect children's mental health (S. Weine et al., 2014), as minors living in supportive familial environments have demonstrated better resilience (Daud et al., 2008; Pieloch et al., 2016). As a result, a large body of research highlights the necessity for interventions targeting parental traumatic distress to improve the mental health of refugees' and asylum seekers' children (Akesson & Sousa, 2020; Eruyar et al., 2018, 2020).

The integrated group parenting intervention, Learning Through Play (LTP) Plus Eye Movement Desensitization and Reprocessing Group Treatment Protocol (EMDR G‐TEP), has been designed to address parental mental health and improve the healthy development of children (Kaptan, Varese, et al., 2021). LTP + EMDR G‐TEP has two components: Component 1 consists of LTP, which is a ten‐session group parenting intervention. LTP aims to promote healthy child development by improving parents' mental health and strengthening attachment between parents and their children (The Hincks‐Dellcrest Centre, 2002), and is an intervention informed by theories of attachment and cognitive development. The manual covers the important aspects of child development by focusing on broad areas, including physical development, cognitive development, psychological development, interpersonal relationships, and communication. The key feature of LTP is the pictorial calendar which highlights the importance of parent−child play. The calendar covers child development from birth until 3 years and helps parents to understand how attachment works. The calendar also contains culturally adapted illustrations of age‐appropriate activities which parents can engage in with their children at home to strengthen the parent−child attachment bond. The program can be easily delivered to disadvantaged groups as it does not require participants to possess formal education. The LTP program was developed in Canada and has been evaluated in several trials. In a randomized controlled trial (RCT) with depressed mothers in Pakistan, Husain et al. (2017) observed significant reductions in depression and parenting stress, which were maintained at the 6‐month follow‐up assessment. These findings have been confirmed by further RCTs with mothers of undernourished children (Khan et al., 2019) and depressed fathers (Husain et al., 2021).

The second component of the integrated intervention is EMDR G‐TEP (E. Shapiro, 2013). EMDR G‐TEP is a group psychotherapy intervention for adolescents and adults who experience various forms of psychological distress as a result of potentially traumatic life experiences. The group protocol covers the core principles of standard individual EMDR therapy (F. Shapiro, 2007) with a special focus on stabilization and positive future resources. The G‐TEP protocol has been tested in several studies with promising results for the treatment of PTSD, depression and anxiety (Kaptan, Dursun, et al., 2021; Lehnung et al., 2016; Roberts, 2018; Tsouvelas et al., 2019; Yurtsever et al., 2018).

This paper reports the pretrial qualitative findings from interviews with refugees, asylum seekers, and professionals working with these groups. This study aims to: (a) identify recommendations for successful engagement and recruitment; (b) gain insights into the issues that might pose barriers to delivery, as well as potential solutions; (c) explore their perceptions of LTP + EMDR G‐TEP intervention; (d) assess needs and interests of the sample group.

To the best of our knowledge, this is the first pretrial qualitative study focusing on a parenting and EMDR G‐TEP intervention, and reporting qualitative findings on the design of the intervention.

## 2 METHODS

We have used the Consolidated Criteria for Reporting Qualitative Research (COREQ) (Tong et al., 2007) in reporting our work to ensure transparency. The checklist consists of 32 criteria that covers different aspects of qualitative studies of related to recruitment, data collection, data analysis, and reporting of the findings.

### 2.1 Study design

This study is a qualitative study using one‐to‐one semi‐structured interviews with two participant groups: (a) adult RAS; (b) professionals who work with the first group. This method was chosen to identify potential barriers and facilitators to implementing the planned intervention. Ethical approval was obtained from the University of Manchester, Research Ethics Committee (Ref: 2020‐7904‐13152).

### 2.2 Sample

Snowball and purposive sampling strategies were adopted to recruit participants, which involved contacting relevant charities and organizations. This included leaflets, telephone calls, and e‐mail invitations. We also used existing links between the first author (S. K. K.) and the target groups. Inclusion criteria for the refugee and asylum seeker group were: (a) aged over 18 years; (b) being a parent; (c) being registered with a GP; (d) ability to speak English. For the professional group, the inclusion criterion was at least 6 months' experience of working with the target groups. See Table 1 for details of the participants.

**Table 1:** Characteristics of participants

Participant number	Gender	Age range	Occupation or employment status	Number of children	Marital status	Working experience with refugees/asylum seekers	Resident status	Time in the UK	Approx**imately** length of interview (min)
Professional 1	M	35−50	Support worker	‐	‐	48 months	‐	‐	66
Professional 2	M	20−35	Psychologist	‐	‐	96 months	‐	‐	68
Professional 3	F	20−35	Mental health practitioner	‐	‐	22 months	‐	‐	38
Professional 4	M	35−50	Mental health practitioner	‐	‐	60 months	‐	‐	52
Professional 5	M	50−65	Support worker	‐	‐	120 months	‐	‐	58
Professional 6	M	50−65	Interpreter	‐	‐	84 months	‐	‐	72
Professional 7	F	35−50	Support worker	‐	‐	60 months	‐	‐	56
Professional 8	F	20−35	Mental health practitioner	‐	‐	60 months	‐	‐	81
Professional 9	M	50−65	Support worker	‐	‐	15 Years	‐	‐	70
Professional 10	F	35−50	Mental health practitioner	‐	‐	9 months	‐	‐	55
Professional 11	M	35−50	Support worker	‐	‐	30 Months	‐	‐	57
RAS 1	F	20−35	Unemployed	1	Separated	‐	Refugee	18 months	53
RAS 2	F	35−50	Unemployed	4	Separated	‐	Asylum seeker	60 months	60
RAS 3	F	35−50	Employed	3	Married	‐	Refugee	36 months	55
RAS 4	F	35−50	Unemployed	2	Married	‐	Asylum seeker	60 months	40
RAS 5	M	35−50	Employed	1	Separated	‐	Refugee	36 months	52
RAS 6	F	35−50	Unemployed	1	Married	‐	Refugee	13 months	45
RAS 7	M	35−50	Employed	1	Married	‐	Asylum seeker	17 Years	37

Abbreviation: RAS, refugees and asylum seekers.

### 2.3 Data collection

Interviews were performed in 2020 by the first author in a private room at the participants' chosen venues or via online platforms. The first author, S. K. K., is a PhD candidate at the University of Manchester, in a Clinical Psychology program. Before the interviews, S. K. K. explained his role, his interests, and the aims of the study. The interviews were conducted in English, audio‐recorded, and transcribed verbatim by the first‐named authors. Two different semi‐structured topic guides, one for RAS, and one for professionals, were developed based on previous works. The topic guides were also informed by a temporal parallel purpose framework (Maher & Neale, 2019). This framework offers a structure for designing an informative qualitative study to improve the design of the intervention. The topic guide included issues with the content of LTP and EMDR G‐TEP interventions, their delivery, perceived acceptability, feasibility, and barriers and facilitators to participation. Before the interviews commenced, participants were given a summary of the treatment manuals. Moreover, the steps involved in the interventions were demonstrated to participants before the interview, and during the interview if necessary. Participants were requested to summarize their understanding of the intervention before they answered any of the interview questions to make sure that they had understood it. Interview length ranged from 38−81 min (mean = 56), exclusive of the time spent on demonstrating the treatment materials. All participants gave written informed consent. The topic guide was pilot‐tested and continuously updated as the interviews proceeded. Transcriptions of the interviews were checked against the audio recordings.

### 2.4 Data analysis

As the aim was to explore the perceptions of the participants along with the barriers and facilitators in delivering the intervention, the interviews were transcribed verbatim and analyzed using thematic analysis (Braun & Clarke, 2006). The analysis involved six steps, starting with familiarization with the data and initial coding and proceeding through to organizing the initial codes into themes, which were named in step 5 and written up in step 6. Data were coded by the first author using the NVivo software, and transcriptions were double coded by other authors to generate themes and sub‐themes, which were reviewed and updated through discussion. Despite previous literature suggesting that data saturation in the thematic analysis might be reached with 12 participants (Guest et al., 2006), the current study deployed data saturation according to the latest debates on the topic (Braun & Clarke, 2019; O'Reilly & Parker, 2013).

## 3 RESULTS

### 3.1 Sample

A total of 20 individuals were invited to participate in the study, of which 18 expressed a wish to do so. Of these 18 people, 11 were professionals and 7 were refugee or asylum seeker parents. Among the professionals, seven participants (63%) were male and four were female (37%), while among the caregivers, five (71%) were female. Regarding marital status among the parents, four (57%) were married and three (43%) were separated. Of the seven parents, four of them had one child, and three had two or more children. As shown in Table 1, on average the parents had been in the United Kingdom for 61 months, with a range from 13 months to 17 years. Four parents had refugee status, while more than half of them (*N* = 4) were unemployed. Finally, among the professionals, five were mental health professionals and the average length of experience of working with RAS was nearly 70 months. See Table 1 for full details of the participants.

### 3.2 Themes and sub‐themes

**Figure 1:**
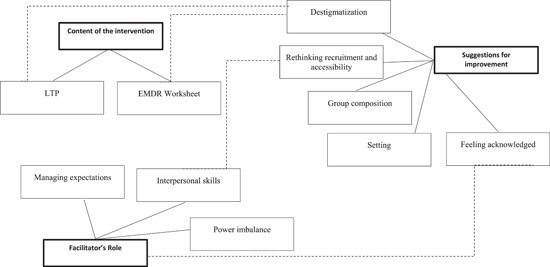
Thematic map. LTP, Learning Through Play.

We generated three main themes through data analysis. These were: (1) intervention perceived as positive; (2) suggestions for improvement; (3) understanding the role of the facilitator. We also generated subthemes and included quotes to support the themes. See Figure 1 for a thematic map.

#### 3.2.1 Content of the intervention

The first theme encompasses the perceptions of the intervention content as understood by the participants.

##### EMDR G‐TEP worksheet

Many of the participants shared their views about the EMDR G‐TEP worksheet. The positive perception of the G‐TEP worksheet and its advantages were evident throughout the interviews. Participants acknowledged several strengths of the intervention, including the benefits of the activity‐based nature of the worksheet. In addition, participants indicated a general consensus on how important it is to not share traumatic experiences during the sessions. Using the EMDR G‐TEP worksheet seemed to improve issues of privacy which have been identified as a significant barrier to seeking mental health treatment amongst RAS (Byrow et al., 2020).

P 11: I think this method (G‐TEP worksheet) which takes away the person to person speaking and talking about their problems is a good thing. I think you have to make it clear that it's not group therapy in the sense that people can talk to each other. It's that they are in the same room but everybody's doing their worksheets.

Despite its critical importance in healing, the expressing or sharing of emotions is a challenging phenomenon among displaced individuals, as many RAS may show reluctant behaviors in talking about their difficulties or emotions (Shannon et al., 2012). In line with the previous point, several participants reported how they consider the G‐TEP worksheet to be helpful against lack of willingness or difficulties in expressing emotions among the target group. These participants valued the flexibility of the worksheet as it allows participants to use drawings or symbols when expressing traumatic experiences.

P 2: The first thing is it is illustrated (EMDR G‐TEP worksheet), every step is there; this is one point. The second thing, you do not need to say anything; you need just to draw, because sometimes, you know, there are people who don't know or don't want to express themselves. It's very comfortable because you don't need to tell anybody about your problem, and you just draw it, maybe in a symbolic way.

The value of having EMDR therapy in a group setting, compared to individual therapy, was also acknowledged by several interviewees. Some participants valued the idea of the group as, to them, group work creates a feeling of security, of safety nets, and sets the stage for therapy.

RAS 1: Often when you're on that stage you don't, or you won't be able to communicate with others. And then you have one on one therapy. You only talk about yourself, and you are in a place that you are forced to talk about them. And you do not want to talk about them, because the more you go deep into them, the more you're just digging them. I find it really difficult. So, I would say this worksheet will give them a safer space.

Some participants discussed the design and layout of the G‐TEP worksheet. These included several facilitators that could improve the participation and attractiveness of the intervention. Several participants indicated the advantages of the worksheet in addressing other challenges related to different understandings of mental health, which mirrors the relevant literature (Farley et al., 2014; N. K. Jensen et al., 2013).

P 11: I think the design of it is inviting for people to participate, and it's the layout and colouring. It's also soothing in a sense.

P 2: I think it is very useful. Because the first thing, it is illustrated (EMDR G‐TEP worksheet); every step is there, and it is quite helpful.

However, some participants highlighted several caveats indicating a potential communication challenge that might put participants off. These participants suggested simplifying the language of the EMDR G‐TEP worksheet as the main strategy to overcome linguistic challenges. This was specifically recommended when working with participants from non‐western cultures. Other language‐related recommendations included using local terminology on the worksheet.

P 10: I think this worksheet looks quite complex at first. Okay, so what is this? To explain it verbally, and to go through it, and I think people will spend some time; I mean, refugees or asylum seekers. You know, you're asking people to do something quite strange to most people. So, to explain a little bit about how it might work might be useful.

P 11: … for example, the word “trauma.” I noticed some words used in psychology, handbooks, or textbooks. Nobody uses them in everyday language; for example, nobody uses the word “trauma” in a refugee camp.

##### Approaches to parenting and LTP manual

Participants acknowledged that the parenting component of the intervention may make the intervention more acceptable and accessible, as it can remove the stigma around receiving mental health support, which was considered as a barrier throughout the interviews.

P 9: I think parenting is quite popular. It is probably more attractive. I mean, even though your training is mental health‐related, if you use the parenting route, I mean, if you are using the parenting as a kind of first stage, engagement will be higher.

P 6: To be honest they love parenting interventions more. Because to them, children are vital. It will attract their attention more if you label this as a parenting intervention.

Assisting participants to overcome their parenting challenges or to accommodate their parenting needs were seen as vital aspects of improving responses to mental health challenges. It was also stated repetitively that parent refugee and asylum seeker parents seek help to become better parents for their children and that the wellbeing of their children is a priority for them.

P 5: So they've got a lot of PTSD or disturbing memories that they are trying to manage. What you quickly discover is that, for a lot of these people, there is the wellbeing of their children that is worrying them a lot. And if that is not to be addressed, their anxiety levels would not drop.

P 11: Everybody has problems with their kids; but refugees, asylum seekers, if you ask them about parenting, I think this is the number one problem that occupies their minds. And it becomes more urgent and more nagging as they grow up.

Participants also praised the LTP manual and its pictorial calendar, as they found it to be culturally adapted.

P 18: It is a tailored programme for people from certain backgrounds, let's say ethnic backgrounds, and delivered by a person also from their community, their heritage culture. I think this will help people to have more trust in the content of this programme.

#### 3.2.2 Suggestions for improvement and implementation

The previous theme encompasses how participants portray both components of the intervention and how their perceptions might inform it. The following theme covers the importance of a culturally and personally adapted approach derived from participants' personal and professional experiences. Moreover, the theme includes how these might tailor the delivery and the content of the interventions to remove stigma, specifically through activities and wording.

##### Destigmatization strategies

The stigma surrounding mental health in RAS was well pronounced by participants and has been well documented in the previous literature. Around half of the participants emphasized the impact of appropriate language in increasing participation when introducing the intervention. To serve this goal, participants noted several recommendations. They described struggling with wording, and how positive wording is linked with hope for the future and might increase engagement by improving the fit of the planned intervention. They also stated that rebranding the intervention, for example as “wellbeing,” “skills training,” or “parenting,” might encourage individuals to participate in this study as it avoids highly stigmatized terms such as “mental health” or “therapy.”

P 1: Basically, if you approach them from a mental health perspective, it's less likely for them to get involved. But if you approach them from that positive side, so the idea is to have a better future or a more hopeful kind of approach, it is more likely for them to get in involved.

P 5: What I think the best option which will improve their participation is, if you remove “therapy”; do it, but say we are providing learning support or training.

More recommendations were offered to improve the acceptability of the intervention. Participants indicated that interventions that are activity‐based improve the attendance and are linked with reduced stigma and increased acceptability in the context of displaced groups. One participant described their practice as an example.

P 8: I think there is stigma and shame, but not so much when they come to see me or when they come to see my colleagues; I think my understanding from the community is that they don't perceive my work as proper therapy, because I'm doing a lot of fun stuff and activity.

P 4: Because it's interactive, so they might buy it, get more interested in doing it. Okay, and if it is not interactive, for example, someone is talking, talking, talking, and doing nothing during sessions, they do not understand the layperson's perspective.

Participants described how the support of partners or other family members can be encouraging, acknowledging that many participants were satisfied with the support received from family members.

P 1: Okay, I don't have this idea that refugee husbands or wives are not open to their partners going to treatment. In many cases… I have many cases that they have been supportive of each other and their children in going and looking for mental health support from the GPs or the health care services. I do not remember any experience of people preventing their partners or children going or not taking care of their children if they need a medical health intervention.

##### Rethinking recruitment and accessibility

Almost none of the participants were supportive of the use of leaflets or social media as a means of recruiting. Word of mouth‐type forms of advertisement were felt to be more compatible with the characteristics of the target groups. Moreover, many of the participants expressed the opinion that it is better for the invitation to come from a person that they were already in contact with.

P 1: Even though I work with refugees, with those who are educated, also for all of the people coming to our service, it simply is just through word of mouth. So, they do not go to read the leaflets. Even though they can read English, they do not pay attention to the leaflets, because I think they are not used to it.

RAS 1: So how did I get involved in different charities or organizations that helped me to be feeling more alive and useful, was the chain like one person could have advertised it to others, and they could tell others “please join, it's very interesting” so and so.

Explaining the study in person to potential participants was linked to building initial rapport with the group. A participant also mentioned the use of “taster sessions” as an effective form of recruitment.

RAS 5: Most of refugees and asylum seekers want to know you or believe you. They want to see what this is. So, once you go to them, they can understand and believe you: “Oh, this is what they are saying, and it makes sense.” So, if you book an appointment, I think if you go to them to have a chat, or if you go to their drop‐in sessions, I believe if you go there, you can talk to them, every one. So, I think it will be better than the internet.

Several participants also suggested that charities and other organizations were better platforms for creating a safe space and gaining trust, which might lead to normalization and a better acceptance of the planned interventions. This was echoed by other participants who mentioned that expressing mental health difficulties came only after they had spent some time with the services.

P 11: So yes, going through intermediaries, agencies or charities makes it easier to find people and meet them. And at the same time, you can win more trust from them based on the trust they have in these charities. And eventually, after some time, or once you develop a relationship, they might open up and say, “actually, I don't feel well”.

##### Group compositions as a prerequisite

The importance of appropriate groupings within the sample was highlighted. Several participants implied that forcibly displaced individuals are not a single, homogeneous group, and different sub‐groups might have unique needs and requirements.

P 9: Once you are with asylum seekers, they are starting to complain about how difficult life is. But refugees do not want to listen to those (complaints) because they passed that period. It reminds them of the situation that they have passed, and they try to get a new life rather than continuing reflecting on that. So, the only negative point that they most often see about our project is we have this mix of groups, refugees and asylum seekers.

The grouping of participants should match their preferences since differences at the levels of language, legal status or the period of time they have lived in the host country might impact their attitudes to, and readiness for the planned intervention. Some participants reported the benefits of being in same‐gender groups as a means of sharing challenges and feeling freer to talk.

P 2: I think you should create two groups. The only criteria we usually use is their English level. One other important part is the number of years they have been in this country.

RAS 4: If I am with another lady or another woman, I am more stable than speaking alone. So, what I am seeing, like if I tell her I have this pain, she knows what I mean. But man, they give lots of descriptions. So, for a woman, we are more comfortable among other women because I know they know that experience.

##### Setting

Several participants noted that their previous experiences might remind them of previous traumatic experiences and shape their mindsets for future treatments. Reflecting on that, the following participant voiced the idea of using outdoor settings to remove the feeling of entrapment.

RAS 1: I would say not in an old or traditional therapy room; I would say nature, like beside a river, or in a mountain, a place that you can feel gathered, like camping, all people gather together and you are in nature; you feel like you're not trapped, you're not imprisoned. You feel more connected to the, to the moment being in passing and being involved; then you feel like you must go to therapy. You have to go to the room; there are two seats in front of each other, you know; in your mindset that looks more like an interrogation than intervention.

Due to issues including stigma and confidentially, the setting/venue of the intervention emerged as fundamentally important. Many participants suggested holding the sessions in a setting where trust is already established with the participants, or somewhere they would feel comfortable. A familiar setting was seen as an icebreaker as it removes shame along with logistical and practical barriers.

P 10: Use a place that people would use anyway so that they would be familiar with it and, that is, that seemed good because people know where it is and they are comfortable to come there, and this is not out of people's comfort zone anyway, so the place that people come in is within their comfort zone. And that makes it, I think, slightly easier.

RAS 7: I believe in an organization where we know the other people. If I go there, I have lots of friends around. So, I am not ashamed to do anything because I have my friends around, which will give me the confidence to ask silly questions that I really want…. If you just invite me somewhere I do not know, I mean I will not be able to have that feeling, you know. I will withdraw because I am not so used to you and that place.

Although many participants suggested delivering the planned intervention at places where communities already have easy access, some participants raised fears about confidentiality due to close networks that exist in the communities. As a solution, many participants suggested that the place should be “invisible” to allow confidentiality.

P 8: It should be confidential and safe. I mean, if it is possible; I do not know how it works, but if it is possible to keep the room or the building closed for the duration, then it might actually be a good place, because they would be able to sort of allow their thinking.

##### Feeling acknowledged and supported

Many participants mentioned the distinct characteristics within refugee and asylum seeker communities, and between refugees, asylum seekers, and dominant Western cultures, in the conceptualization of mental health and treatments. Participants recommended that any intervention targeting mental health or parenting skills should understand the cultural background of RAS, which suggests tailored content without imposing culturally insensitive conceptions.

P 1: The point is refugees are not a homogeneous community. It depends on who they are, their background, their country, their culture or their level of education. But, like I said, it depends on whom you are approaching, what segments of refugees you are approaching, and then you need to decide based on the refugees that you have access to; you need to tailor your content to them.

The culturally sensitive approach was echoed by another participant who suggested not presuming that there is a universal way of being a parent. The presumption of a universally accepted way of parenting might be a barrier to recognizing cultural differences, therefore preventing participants from accessing interventions.

P 13: There are many ideas that have been developed in Europe, or what the good mother is supposed to look like and what the good father is supposed to look like, those that were developed around specific ways of living, whether that's financial, well, it's quite complex. What is healthy here may not be unhealthy in their culture. Yes. So, it is mainly around the understanding.

Many participants appreciated interventions that would be compatible with participants' current living status, expectations and needs, as an absence of such understandings within interventions were seen as a barrier. They mentioned that an acknowledgment of the needs of the participants acted as a key to long‐term engagement.

P 4: We try to see what they need, for example most of them say, “we want to have a driving license.” So, our English classes are designed on having the driving licence, and they are totally engaged in the content. But if they go to, for example, English for Speakers of Other Languages, which is about travelling to Barcelona or having your holiday in somewhere, it is not cultural, and it is not really about what they need, so they are not engaged.

#### 3.2.3 Understanding the role of the facilitator

This theme addresses how participants define the role and motivations of facilitators, and how it impacts participants' willingness to take part. This included several recommendations for the training of facilitators. Moreover, several participants indicated prominent issues about the facilitator's role, which ranged from their personal capacity to more salient behaviors of respect and interpersonal skills.

##### Power imbalance/savior complex

Some participants raised concerns around the role of the facilitators and the way facilitators perceive participants. They suggested that perceptions of participants as damaged and broken might compromise the therapeutic relationship and create pressure for participants.

P 7: People might have quite a bit of a saviour complex. I think it comes from probably a very good place. They [public] want to help people whom they perceive need help. And I think it can be quite damaging if you only see people as damaged and broken, then that it is your job to fix them.

In addition, the concept of power imbalance emerged from the interviews as an important behavior to be mindful of in terms of the involvement of participants. Some participants expressed concerns about some participants who always agree with the facilitators/professionals. The following quote suggested that appropriate interaction with participants is an important domain in the training of facilitators.

P 8: So if there is something to be said about the power that we professionals have and the authority over this client group; for example, some clients that I've worked with, they will do everything that I ask, because they think I have professional authority and they must listen to me.

##### Managing expectations

A message that needs to be conveyed to the participants before the intervention is the power and capacity of the group leader. When asked to consider the facilitators' role, participants indicated that the research team/facilitators should clarify their capacity, role, aims and professional boundaries to avoid unrealistic expectations.

RAS 6: The group leader or someone can help them in everything; like, they can even bring their family from their countries, like you have that power.

Some participants also expressed concerns regarding professional boundaries. They also discussed how some attitudes contribute to creating dependency. Attention to facilitator training and recognition to respond to such behaviors was recommended.

P 7: If you are trying to, if you are maybe a befriender, you might be asked to do things that are outside your role. If people say, “oh, I am struggling, if you got any money” or, you know, “if you got any food” and that might not be appropriate to ask because it creates dependency.

Participants described several factors affecting their trust and their willingness to talk about mental health difficulties. For some participants, expressing mental health difficulties came only after they had spent some time with the services.

P1: Because once we start supporting families or individuals, after one month or two months, then we have that relationship; we can have a good understanding of their situation; they have that trust in our service.

P 9: Eventually, after some time, or once you develop a relationship, they might open up and say “actually, I don't feel well.”

Participants also addressed beliefs, including distrust of authorities, which might result in reduced engagement, discomfort and fear of disclosing information. This was specifically linked with asylum seekers' concerns over their residence status.

P 7: I think there is always a fear, or they are not always keen on talking to people with authority or trying to ask the personal details. They think everything is related to the government in one way or the other.

P 10: People have come from a really troubled place and troubled times, and there's a sense of “why would people trust me,” and I understand that, like “what are you going to do with my data?” And where is this going? Will this affect Home Office decisions? Or like, how much power I got as a researcher, and just so; that is a massive thing.

##### Valuing interpersonal skills

Participants emphasized that they found the feeling of acceptance appealing, meaning this improved their engagement with the intervention. Many participants also said that the characteristics of the facilitator are very important for engagement with the intervention.

RAS 3: I think, as human beings, we just want to run after the solution; we could make life better if they say…, if they tell us on that situation; you could have this, you could have done this, and your life would have changed. I want to say “no, no, I did the right thing,” even though I knew I made a mistake.

RAS 2: Whatever they (RAS) do, you should just reassure them that they are not the only one, I mean, with problems. Whatever they say, you believe them; you may not know what they are saying but say you believe them.

## 4 DISCUSSION

Several studies have showed that parenting interventions hold promising results (Ballard et al., 2018; El‐Khani et al., 2018). However, there is a lack of knowledge about what works in recruiting and maintaining attendance among refugee and asylum seeker parents. This gap in the field has been echoed by previous studies (Sadavoy et al., 2004; Thomson et al., 2015), which refer to specific exploration of the views, barriers and facilitators associated with proposed interventions to improve access (Lakkis et al., 2020; Sim et al., 2019). Therefore, this pretrial qualitative study is aimed at adding to the existing literature by exploring factors that might influence recruitment for, delivery of, and engagement with the planned multicomponent intervention.

We conducted a total of 18 interviews with refugee and asylum seeker parents, and professionals who work with these two groups, to explore their perceptions to develop an acceptable intervention. The findings highlight several themes regarding personal and cultural preferences, methods of destigmatization, treatment content, group composition and practical issues, including the role of facilitators and how to address barriers.

What did we learn from this study? Past studies have reported the need for multicomponent treatments that address not only parenting needs, but also mental health difficulties in a family context (Miller et al., 2020; Moran & Ghate, 2005). In agreement with this, it appears to be feasible to deliver the EMDR G‐TEP manual integrated into the LTP parenting intervention to keep parents engaged in the intervention. The parenting component with its positive language was found to be a useful destigmatization strategy. Moreover, culturally adapted parenting manuals were found to be engaging and related to the target group's needs. Additionally, as quoted above, the pictorially‐presented and colorful G‐TEP worksheet was received very favorably. The interactive nature of the G‐TEP intervention was also praised by participants, since it is suggested that the target group is less likely to engage in one‐way psychoeducation programs in which they remain inactive (Moran & Ghate, 2005). This was also acknowledged as another strategy for destigmatization. However, some participants made suggestions for simplifying the language of the G‐TEP worksheet.

Several articles have reported the importance of culturally and personally sensitive services to improve access to care (Byrow et al., 2020; Salami et al., 2019; Satinsky et al., 2019). In line with previous literature, we find that the context in which the target groups lives, their needs and previous experiences might function as barriers to participating in mental health and parenting trials. This suggests specific exploration of the factors related to treatment setting, group composition, language, and other personal preferences when delivering interventions. Findings from previous studies have also showed that stigma and shame around mental health is an important factor that prevents RAS from accessing health care services (Clement et al., 2015; Robertshaw et al., 2017). Consistent with this, our analysis shows the importance of providing care that is mindful of such challenges, as many of the participants mentioned that these, especially stigma, were the most common reasons that might prevent them from taking part in mental health‐related interventions. However, the findings of the current study also suggest several solutions to overcome these challenges by offering several destigmatization strategies. These include using positive wordings, such as “life‐skills training” or “personal development,” and the elimination of the phrase “mental health.” Such a rebranding of mental health interventions was felt to destigmatize participation and reduce shame, which makes the intervention more acceptable for parents and communities.

Challenges in ensuring confidentiality or establishing trust with refugees or asylum seekers have been widely acknowledged by the previous literature (Colucci et al., 2015; Robertshaw et al., 2017). Many studies have mentioned that there is a fear of disclosure due to residency status or the level of mistrust towards professionals (Bhatia & Wallace, 2007; Feldmann, Bensing, de Ruijter, & Boeije, 2007; Haith‐Cooper & Bradshaw, 2013). In line with this, many participants stressed that the EMDR G‐TEP manual may give space for personal confidentiality and allay concerns about this, as the manual does not require participants to share their traumatic experiences with the rest of the group.

Several other recommendations were also highlighted by p'articipants in terms of improving access to services. Findings reveal that group composition, the setting and the facilitator are important for participants before they decide to take part. For example, the use of leaflets as a means of advertisement was not supported, while many participants suggested that charities and organizations trusted by RAS could offer brief taster sessions. This was associated with a high level of trust in such organizations as it removes distrust of authorities, which is well known in the field (Gross et al., 2001).

We also learned that groups with mixed residence status (RAS) may also depress attendance rates. Instead, we were advised to establish separate groups based on residence status and gender to make the groups more homogenous and more targeted. Additionally, some participants suggested a further criterion, years spent in the host country, when grouping participants. In terms of the characteristics of facilitators, the findings raise important points as to how the role of facilitators and the setting of boundaries between participants and facilitators are essential. In line with past literature (Milner, 2015), in cases where facilitators perceive themselves as “saviours” of “broken and damaged people,” the quality of the relationship with facilitators would be diminished off‐putting for participants. Thus, the importance of ensuring awareness of boundaries and of the limits of facilitators before the sessions was acknowledged. This includes several examples, such as what participants can ask of a facilitator, or the kind of help they can expect to receive.

Finally, the current findings once again reveal the widely studied stigma surrounding mental health. Thus, we have decided to rebrand our intervention and to use the term “wellbeing training,” rather than “mental health intervention” to make it more friendly, as suggested by the participants. We have also decided to emphasize the parenting component which, along with simplified mental health terminology, may make the intervention more acceptable. Finally, to make participants familiar with the terminology and layout of the G‐TEP worksheet, we have added mini EMDR G‐TEP practices into all sessions.

## 5 CONCLUSION

What does this study tell us? First, there have been several advantages to devoting time to this pretrial qualitative study as it supports the use of the LTP + EMDR G‐TEP intervention. Second, it has provided us with useful information for the design of the proposed intervention by offering several modifications which might improve its acceptability and accessibility.

One strength of this study is that it includes both professionals and individuals from target groups while following an established framework for the written topic guides (Maher & Neale, 2019). Moreover, the snowball and purposive sampling methods were useful in allowing a wide range of disciplines to be covered. Finally, we believe that the respectable level of involvement by professionals will help us to transfer our findings to the real world. The study has also several limitations. The participants were recruited from local areas in Manchester, so the findings may not be generalizable to other settings. Moreover, due to budgeting problems, we only recruited participants who are registered with GPs and who can speak English. We accept that the sample might not represent other demographic groups who either do not have access to a GP or are unable to speak English.

## AUTHOR CONTRIBUTIONS

All authors contributed in terms of conceptualization, methodology and writing. Safa Kemal Kaptan, Betul Yilmaz, and Panoraia Andriopoulou participated in data collection and data analysis. Nusrat Husain, Filippo Varese, and Panoraia Andriopoulou contributed to manuscript preparation and review. All authors have read and have agreed to the published version of the manuscript.

## CONFLICT OF INTEREST

The author declare no conflict of interest.

### PEER REVIEW

The peer review history for this article is available at https://publons.com/publon/10.1002/jcop.22908

## ETHICAL APPROVAL

Ethical approval was obtained from the University of Manchester, Research Ethics Committee (Ref: 2020‐7904‐13152). Informed consent was obtained from all subjects involved in the study.

## Supporting information

Supplementary information.

Supplementary information.

## Data Availability

The data that support the findings of this study are available from the corresponding author upon reasonable request. The data from the current study are available from the corresponding author on reasonable request.
