# Supplementary material for: What works? Lessons from a pretrial qualitative study to inform a multi‐component intervention for refugees and asylum seekers: Learning Through Play and EMDR Group Traumatic Episode Protocol
Source: J Community Psychol. 2022 Jun 14;51(1):361–81. doi: 10.1002/jcop.22908 (PMC10084026; doi:10.1002/jcop.22908)
Supplement: Supplementary file 1 — Supplementary information. [file JCOP-51-361-s002.docx]

**Topic Guide for Refugees or Asylum Seekers**

**Preamble**

Thank for agreeing to take part in this interview.

Introduce the study and aims of the interview.

Answer questions of participants

Get the Consent Form signed

**A. General Questions**

1. Do you think refugees or asylum seekers seek mental health or parental help? If so, who/where from?

**Prompts**: What might be the reasons or barriers to accessing help/treatment? What could be done?

1. What challenges do you face while seeking mental health or parental help? What can be done in response to these challenges?

**Prompts**: Transport issues, family pressure, religion, cultural discrimination or stigma, feelings towards ‘western-style medicine’.

1. What would your family/community think if you attend mental health or parental training?

**Prompts**: Do they think less of you? Gossip or speak badly about you? Would their relationships with you change?

1. Do you think your partner would be ok with your going for mental health or parental training? What makes them comfortable/uncomfortable?

**Prompts**: What conditions would make them more supportive? Female/male therapist, location.

1. Whose ideas are important for you / can encourage you when you decide to receive mental health or parental support (political, religious, family leaders)?

**Prompts**: How can leaders encourage you? Which leaders would be best able to encourage? What can be done to help convince leaders to encourage people?

1. If training was available in a refugee community, what are the best strategies to advertise a new program?

**Preamble**

Before asking the questions in group B, introduce Group EMDR Protocol

**B. Questions on Group EMDR**

1. What do you understand by the term “mental health training”?
2. Can you start by telling me your first thoughts about Group EMDR?

**Prompts**: What part of it would be most helpful? What part of it could be difficult or easy to understand? Any aspect that should not be/should be part of training?

1. What might be the reasons that you do not participate in Group EMDR? What can be done in response to these challenges?

**Prompts**: Cultural issues, the content of the intervention, number and duration of sessions.

1. If you were to offer this training what kind of things encourage/discourage you to participate?

**Prompts**: Financial incentives, transportation, domestic duties, fear of community judgment and social stigma, lack of understanding of treatment or treatment complexity, ask recruitment and advertising factors.

1. In your experience, which places might be more suitable for delivering Group EMDR?
2. In your experience, when and how much time are you available for educational activities?
3. Who should facilitate or deliver Group EMDR? (Ask facilitator characteristics)

**Prompts**: Gender or nationality of the researcher, language, affiliations or occupation of

the researcher.

**Preamble**

Before asking the questions in group C, introduce LTP Manual

**C. Questions on LTP**

1. What do you understand by the term ‘parenting training’?
2. Can you start by telling me your first thoughts about LTP?

**Prompts**: What part of it would be most helpful? What part of it could be difficult or easy to understand? Any aspect that should not be/should be part of training?

1. What might be the reasons that you do not participate in LTP? What can be done in response to these challenges?

**Prompts**: Cultural issues, the content of the intervention, number and duration of sessions.

1. If you were to offer this training what kind of things encourage/discourage you to participate?

**Prompts**: Financial incentives, transportation, domestic duties, fear of community judgment and social stigma, lack of understanding of treatment or treatment complexity, ask recruitment and advertising factors.

1. In your experience, which places might be more suitable for delivering Group EMDR?
2. In your experience, when and how much time are they available for educational activities?
3. Who should facilitate or deliver Group EMDR? (Ask facilitator characteristics)

**Prompts**: Gender or nationality of the researcher, language, affiliations or occupation of

the researcher.

Ask if anything else the participant would like to add.
