# Supplementary material for: What works? Lessons from a pretrial qualitative study to inform a multi‐component intervention for refugees and asylum seekers: Learning Through Play and EMDR Group Traumatic Episode Protocol
Source: J Community Psychol. 2022 Jun 14;51(1):361–81. doi: 10.1002/jcop.22908 (PMC10084026; doi:10.1002/jcop.22908)
Supplement: Supplementary file 2 — Supplementary information. [file JCOP-51-361-s001.docx]

**Topic Guide for Service Providers**

**Preamble**

Thank for agreeing to take part in this interview.

Introduce the study and aims of the interview.

Answer questions of participants

Get the Consent Form signed

**A. General Questions**

1. Can you describe your experiences of working with a refugee or an asylum seeker? What and when was it?
2. What challenges do you face, while working with this population? What do you do in response to these challenges? Does working with refugees requires certain skills?

**Prompts**: Time constraints, place of the sessions, transport issues, family pressure, religion, cultural discrimination or stigma, feelings towards ‘western-style medicine’.

1. Do you think refugees or asylum seekers seek mental or parental help? If so, who/where from? What might be the facilitators or barriers to accessing help/treatment? What could be done?

**Prompts**: Families with a parent or child with a disability, young parents, domestic responsibilities.

1. What would their family/community think if they attend mental health or parental training?

**Prompts**: Do they think less of them? Gossip or speak badly about them? Would relationships with the woman or family change?

1. Do you think partners in this community are comfortable with their wives/husbands going for training? What makes them comfortable/uncomfortable?

**Prompts**: What conditions would make them more supportive?

1. If training was available in a refugee community, what are the best strategies to advertise a new program?

**Prompts**: Leaders encouragement? Which leaders would be best able to encourage?

1. How to calm the participants down if at any point during the session they get stressed out?

**Preamble**

Before asking the questions in group B, introduce Group EMDR Protocol

**B. Questions on Group EMDR**

1. Can you start by telling me your first thoughts about Group EMDR?

**Prompts**: What part of it would be most helpful? What part of it could be difficult or easy to understand? Any aspect that should not be/should be part of training?

1. What might be the reasons that they do not participate in Group EMDR? What can be done in response to these challenges?

**Prompts**: Cultural characteristics of the target population, the content of the intervention, number and duration of sessions.

1. What kinds of things might impact their willingness (the acceptability) to take part in Group EMDR?

**Prompts**: Financial incentives, transportation, domestic duties, fear of community judgment and social stigma, lack of understanding of treatment or treatment complexity, ask recruitment and advertising factors.

1. In your experience, which places might be more suitable for delivering Group EMDR?
2. In your experience, when and how much time are they available for educational activities?
3. Who should facilitate or deliver Group EMDR? (Ask facilitator characteristics)

**Prompts**: Gender or nationality of the researcher, language, affiliations or occupation of

the researcher.

**Preamble**

Before asking the questions in group B, introduce LTP Plus Manual

**C. Questions on LTP**

1. Can you start by telling me your first thoughts about LTP?

**Prompts**: What part of it would be most helpful? What part of it could be difficult or easy to understand? Any aspect that should not be/should be part of training?

1. What might be the reasons that they do not participate in LTP? What can be done in response to these challenges?

**Prompts**: Cultural characteristics of the target population, the content of the intervention, number and duration of sessions

1. What kinds of things might impact their willingness (the acceptability) to take part in LTP?

**Prompts**: Financial incentives, transportation, domestic duties, fear of community judgment and social stigma, lack of understanding of treatment or treatment complexity, ask recruitment and advertising factors.

1. In your experience, which places might be more suitable for delivering Group EMDR?
2. In your experience, when and how much time are they available for educational activities?
3. Who should facilitate or deliver Group EMDR? (Ask facilitator characteristics)

**Prompts**: Gender or nationality of the researcher, language, affiliations or occupation of

the researcher.

Ask if anything else the participant would like to add.
